# Spontaneous rupture of spleen in a patient with COVID-19 disease: case report and review of the literature

**DOI:** 10.1093/jscr/rjac124

**Published:** 2022-04-23

**Authors:** Nora H Trabulsi, Sonds S Alshammakh, Alaa A Shabkah, Mohannad Aladawi, Ali H Farsi

**Affiliations:** Department of Surgery, Faculty of Medicine, King Abdulaziz University, Jeddah, Saudi Arabia; Department of Surgery, Faculty of Medicine, King Abdulaziz University, Jeddah, Saudi Arabia; Department of Surgery, International Medical Center, Jeddah, Saudi Arabia; Department of Surgery, Faculty of Medicine, King Abdulaziz University, Jeddah, Saudi Arabia; Department of Surgery, Faculty of Medicine, King Abdulaziz University, Jeddah, Saudi Arabia

## Abstract

Coronavirus disease 2019 (COVID-19) has been associated with multisystemic complications and thrombotic events including pulmonary embolism and deep venous thrombosis. Splenic rupture has been recently reported as a complication in patients with COVID-19, however, the number of cases is limited and the mechanism is still not clearly understood. We present a case of spontaneous splenic rupture secondary to COVID-19 disease.

## INTRODUCTION

Coronavirus disease 2019 (COVID-19) has a wide clinical spectrum, ranging from asymptomatic and mild diseases presenting with lower respiratory tract infection, to severe illness associated with acute respiratory distress syndrome and multiorgan failure [1, 2]. The disease has been reported to have a strong thrombotic tendency which predisposes patients to arterial and venous thrombotic events, however the underlying mechanism has not been yet fully understood [2].

Spontaneous splenic rupture (SSR) is a rare fatal condition with an incidence rate of 0.1%–0.5% [3, 4]. It has different pathological etiologies including neoplastic (30.3%), infectious (27.3%), inflammatory (20%) and drug-related disorders (9.2%) [5]. Recently, COVID-19 has been associated with SSR. We present a case of SSR secondary to COVID-19 and review of the literature.

## CASE REPORT

A 57-year-old male diabetic and hypertensive presented to the emergency department with respiratory distress and was admitted to the intensive care unit (ICU) as a case of respiratory failure secondary to COVID-19 pneumonia. He was transferred to the ward 1 week after stabilization. Few days later, he was found to have bilateral segmental and subsegmental pulmonary embolism (PE) and the patient was immediately started with heparin infusion. The next day, he developed upper limb weakness National Institutes of Health Stroke Scale 4 and decreased Glasgow Coma Scale (GCS) 11, computerized tomography (CT) of the brain was performed and confirmed recent infarction with hemorrhagic transformation.

The patient was also complaining of generalized abdominal pain and vomiting, ultrasound of the abdomen was done initially and showed heterogenous spleen with hyperechoic lesions largest measuring 3.8 × 3 cm likely representing infarcts and mild hepatic steatosis.

**Figure 1:**
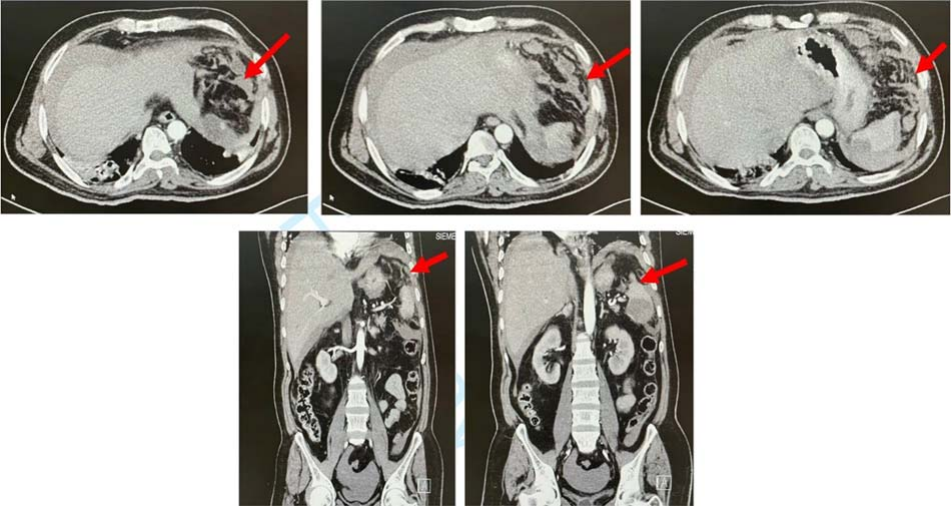
CT abdomen. Axial and coronal views showing splenic rupture and hemoperitoneum (red arrow).

**Table 1:** The clinical presentation and management of reported cases of spontaneous splenic rupture in patients with COVID-19 disease patients

Ref.	Age/Sex	Initial clinical presentation and hospital course	Laboratory and radiological findings	Management	Treatment outcome	Histopathology
Agus *et al.* 2021 [4]	46/F	Initial presentation: abdominal pain and fever. Vitally stable with generalized abdominal guarding.	Hb: 7.7	Definitive management: Laparotomy with splenectomy.	Complicated by wound infection, the patient was discharged on Day 20 after therapy.	Several subcapsular hemorrhages and hematomas. The largest was on the anterior surface of the spleen, compressing the parenchyma leading to splenic rupture.
		Hospital course: the patient deteriorated and became vitally unstable.	CT abdomen: Splenic subcapsular hematoma with hemoperitoneum	Findings of exploration: 2 L of blood and a bulky blood clot attached to the lower pole of the broken spleen.		
Shaukat *et al.* 2021 [11]	57/M	Initial presentation: Cough and diarrhea. He was vitally unstable (hypotensive, tachycardic, hypoxic) with rigid abdomen.	Hb: 7.8	Initial management: Fluid resuscitation and transferred to critical care for ventilatory and circulatory support. He was transfused with packed red cells, FFP and given IV Tranexamic acid.	The patient steadily improved in hospital and was discharged home on Day 24 after therapy.	Not mentioned
			CT abdomen: Hemoperitoneum. The spleen was heterogeneous with evidence of extracapsular rupture.	Definitive management: Splenic artery embolization to stop the hemorrhage, following which he was stabilized.		
Mobayen *et al.* 2020 [12]	52/M	Initial presentation: Abdominal pain and fever. Vitally stable, with tenderness in the epigastrium.	Hb: 11 initially then dropped to 9.5	Definitive management: Laparotomy with splenectomy.	The patient was discharged in a good general condition.	Spleen tissue laceration with microscopic focally hemorrhagic area.
		Hospital course: Patient became febrile and distressed, vitally stable but there was generalized abdominal rebound tenderness.	First abdominal CT: Perihepatic and perisplenic fluid. Second CT Chest / Abdomen: Patchy ground-glass lung lesions with bilateral pleural effusion and extensive fluid around the spleen.	Findings of the exploration: 1 L of blood and broken spleen.		
Knefati *et al.* 2021 [13]	75/F	Initial presentation: Abdominal pain and vomiting	Hb: 12.3	Definitive management: Laparotomy with splenectomy	The patient tolerated the surgery well and recovered without serious complications under ICU care	Area of capsular rupture with an associated area of subcapsular hemorrhage
			CT abdomen: large collection of subcapsular fluid surrounding a small spleen indicating a subcapsular hematoma	Findings of exploration: ruptured spleen with hemoperitoneum		

M: Male. F: Female. Hb: Hemoglobin level in g/dl. ICU: Intensive care unit.

The next day, the patient deteriorated clinically and became hemodynamically unstable. His abdomen was distended with generalized guarding and tenderness. His labs showed leukocytosis and hemoglobin drop from 12 g/dl to 9 g/dl. After resuscitation, CT of the abdomen was performed and showed a large splenic infarct with perisplenic hematoma, contrast extravasation and hemoperitoneum (Fig. 1). The patient deteriorated after CT and became vitally unstable, with low GCS 6, with further drop in hemoglobin to 6 g/dl. The patient was intubated and shifted to ICU for circulatory and respiratory support. He was taken to the operating room and underwent exploratory laparotomy. Upon exploration, 2 L of fresh blood was found in the abdomen, with multiple areas of splenic laceration and rupture. Packing was performed for bleeding control and was followed by splenectomy. Microscopic examination showed areas of extensive necrosis and areas of hemorrhage with blood clots.

Postoperatively, the patient was improving. He resumed his therapeutic dose of heparin 12 h after the operation and was discharged in satisfactory condition. Upon his follow-up visit, he was on a wheelchair as a residual neurological deficit. His wound showed signs of infection that was treated with antibiotics and dressing.

## DISCUSSION

Since the emergence of COVID-19, different nonrespiratory complications including thrombotic events such as myocardial infarctions, cerebrovascular events, deep venous thrombosis (DVT), PE and disseminated intravascular coagulopathy have been reported [6].

The hypercoagulable state in COVID-19 patients is multifactorial and can be explained by the impact of hypoxia and endothelial cell dysfunction causing vasoconstriction, increased blood viscosity and dysregulated immunity. These factors can lead to microthrombosis and predisposition to arterial and venous thrombosis [7, 8]. A Dutch study involving 184 individuals with COVID-19 reported high incidence of thrombotic events (31%) with PE being the most prevalent complication [9]. COVID-19 was also reported to induce microvascular thrombosis and necrosis to the spleen leading to splenic infarction and in some cases subsequent SSR [10]. To date, four cases of SSR secondary to COVID-19 were reported in the literature [4, 11–13]. Clinical and pathological presentation are presented in Table 1.

SSR is considered a fatal condition, early diagnosis and management is necessary to decrease the mortality. Splenectomy is commonly performed as a definitive treatment for this condition, although spleen-preserving procedures are considered in some cases, but it is less frequently performed due to the risk of rebleeding from a fragile spleen. Nonsurgical treatment including arterial embolization can be considered especially in high-risk patients; however, it is usually limited to hemodynamically stable patients [14, 15]. Splenectomy was performed for three of the previously reported cases [4, 12, 13].

Although the precise mechanism of SSR among patients with COVID-19 is still unclear, the presentation of abdominal pain should raise the suspicion of SSR and further evaluation should be performed. As similar cases continue to be reported, COVID-19 might be considered as one of the causes of SSR.

In conclusion, COIVD-19 is a systemic disease with thrombotic tendency affecting different organs. The pathogenesis of SSR is not fully understood due to the limited number of cases. The clinical manifestations and microscopic examination in our case support the phenomena of microthrombosis as one of the underlying mechanisms.
